# Capitellum Fractures in the Pediatric Population: A Case Report

**DOI:** 10.7759/cureus.102820

**Published:** 2026-02-02

**Authors:** Luís B Pinto, Rita F Castro, Luís Fabião, Nuno V Ferreira, Tânia Freitas

**Affiliations:** 1 Orthopaedics and Traumatology, ULS Viseu Dão-Lafões, Viseu, PRT; 2 Orthopaedics and Trauma, ULS Barcelos-Esposende, Barcelos, PRT

**Keywords:** capitellum fractures, elbow surgery, pediatric elbow trauma, pediatric fractures, trash lesions

## Abstract

Capitellum fractures are rare injuries in the pediatric population. Their diagnosis is particularly challenging due to the predominantly cartilaginous nature of the pediatric elbow, which often limits the sensitivity of conventional radiographs. Consequently, these fractures are frequently overlooked at initial presentation.

We present the case of a nine-year-old boy who sustained indirect trauma to the left elbow after a fall from standing height. On clinical examination, the patient complained of pain, mainly in the lateral aspect of the elbow, with refusal to actively mobilize the joint and marked discomfort during passive motion. Initial plain radiographs were inconclusive. Given the high index of clinical suspicion, further evaluation with computed tomography (CT) was performed, which revealed a Kocher-Lorenz fracture of the capitellum (Bryan and Morrey type II). Surgical treatment was undertaken through a lateral elbow approach via the Kocher interval; open reduction and internal fixation of the osteochondral fragment was achieved using bioabsorbable chondral darts. At one-year follow-up, the patient was asymptomatic, had regained a full range of motion of the elbow, and radiographic evaluation demonstrated complete fracture consolidation. No complications were reported.

This case highlights several key aspects of capitellum fractures in the pediatric population. First, it emphasizes the importance of maintaining a high index of suspicion in younger children presenting with elbow pain and inconclusive initial radiographs following trauma. Second, it underlines the crucial role of advanced imaging modalities, particularly CT scan, in confirming the diagnosis and accurately characterizing fracture morphology. Finally, it supports early recognition and surgical management with anatomical reduction and stable fixation as an effective treatment strategy for displaced capitellum fractures. Although larger studies are needed to establish standardized treatment protocols, this case adds to the growing body of evidence suggesting that timely diagnosis and meticulous surgical management can result in excellent clinical and functional outcomes in pediatric capitellum fractures.

## Introduction

Isolated capitellum fractures are an extremely rare entity in the pediatric population, accounting for less than 1% of fractures in children and being even rarer in patients younger than 12 years of age [1-4].

From a mechanical standpoint, capitellum fractures most commonly result from an axial load transmitted through the elbow in extension or slight flexion following a fall onto an outstretched hand (FOOSH). These injuries frequently occur in association with radial head fractures and/or lateral ulnar collateral ligament injuries [1,2,5].

Currently, the most widely used classification system is the Bryan and Morrey classification, which describes these fractures in four types: Type I, or Hahn-Steinthal fracture, characterized by a large osseous fragment of the capitellum that often includes an adjacent portion of the trochlea; Type II, or Kocher-Lorenz fracture, consisting of a smaller fragment composed predominantly of cartilage with minimal subchondral bone; Type III, which includes comminuted capitellum fractures; and Type IV, described by McKee, referring to fractures with a coronal shear pattern involving the capitellum and trochlea [6].

The rarity of these injuries, combined with the anatomical complexity of the growing elbow and the presence of unfused ossification centers, makes diagnosis particularly challenging. Consequently, these fractures are frequently overlooked on initial radiographs. Clinical findings such as ecchymosis, swelling, lateral elbow pain with localized tenderness over the radiocapitellar joint, painful limitation of elbow range of motion, and mechanical block to flexion/extension and/or pronation/supination should raise suspicion of a capitellar fracture. A high index of suspicion and careful clinical evaluation are therefore essential; in selected cases, additional imaging studies with computed tomography (CT) may be required to confirm the diagnosis and accurately define the fracture pattern [1,5,7,8]. For this reason, they have been included among TRASH (The Radiographic Appearance Seemed Harmless) lesions [9].

Surgical treatment is required in the vast majority of cases; the primary goals are anatomical reduction of the articular surface and stable fixation to allow early mobilization and minimize the risk of late complications such as stiffness, osteochondritis, or post-traumatic osteoarthritis [1,2,8,10,11]. Given the rarity of this injury, there is a lack of sufficiently robust studies in the literature to establish well-founded treatment guidelines for capitellum fractures in the pediatric population, particularly in younger children (<12 years). To the best of the authors’ knowledge, at the time of writing, there have been no reported cases of this fracture pattern in such a young patient (nine years old). Therefore, the purpose of this case report is to contribute to the growing body of evidence and knowledge regarding this traumatic pathology, with particular emphasis on diagnostic challenges and surgical management.

## Case presentation

A nine-year-old male patient sustained a fall from standing height, resulting in indirect trauma to the left elbow. He had no relevant past medical history. On physical examination, the patient had swelling and pain over the lateral aspect of the elbow, with refusal to actively mobilize the elbow and guarding during attempted passive motion, particularly with attempted passive forearm pronation and supination.

**Figure 1:**
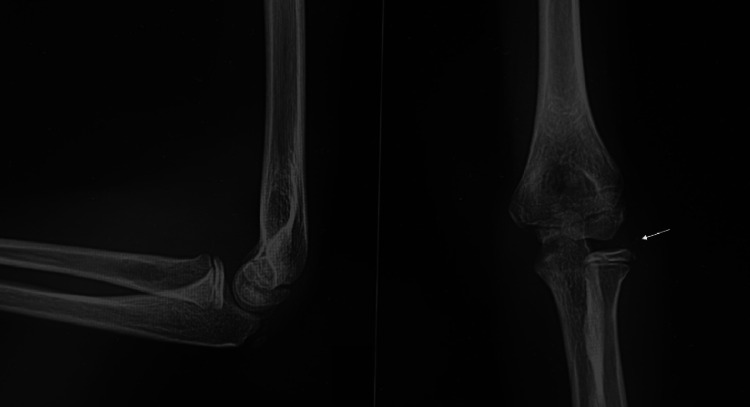
Left elbow radiographs: lateral and anteroposterior views Bony fragments in the radiocapitellar joint (white arrow).

**Figure 2:**
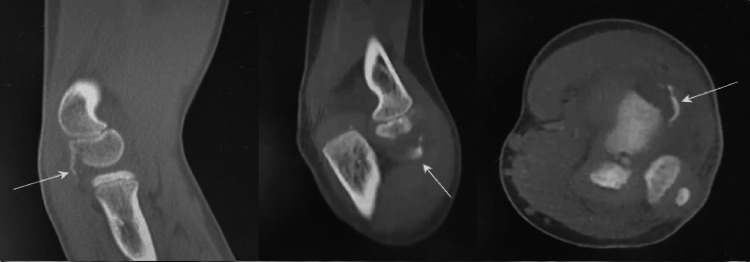
CT scan From left to right: lateral view, posteroanterior view, and axial view showing a Kocher-Lorenz fracture with posterolateral displacement (white arrows).

Conventional radiographs were obtained and did not reveal clear acute traumatic changes, except for the presence of a positive fat pad sign and small osseous fragments within the radiocapitellar joint. Given the high index of clinical suspicion, a CT scan was performed, which demonstrated a Kocher-Lorenz fracture (Bryan-Morrey type II) (Figures 1-2).

**Figure 3:**
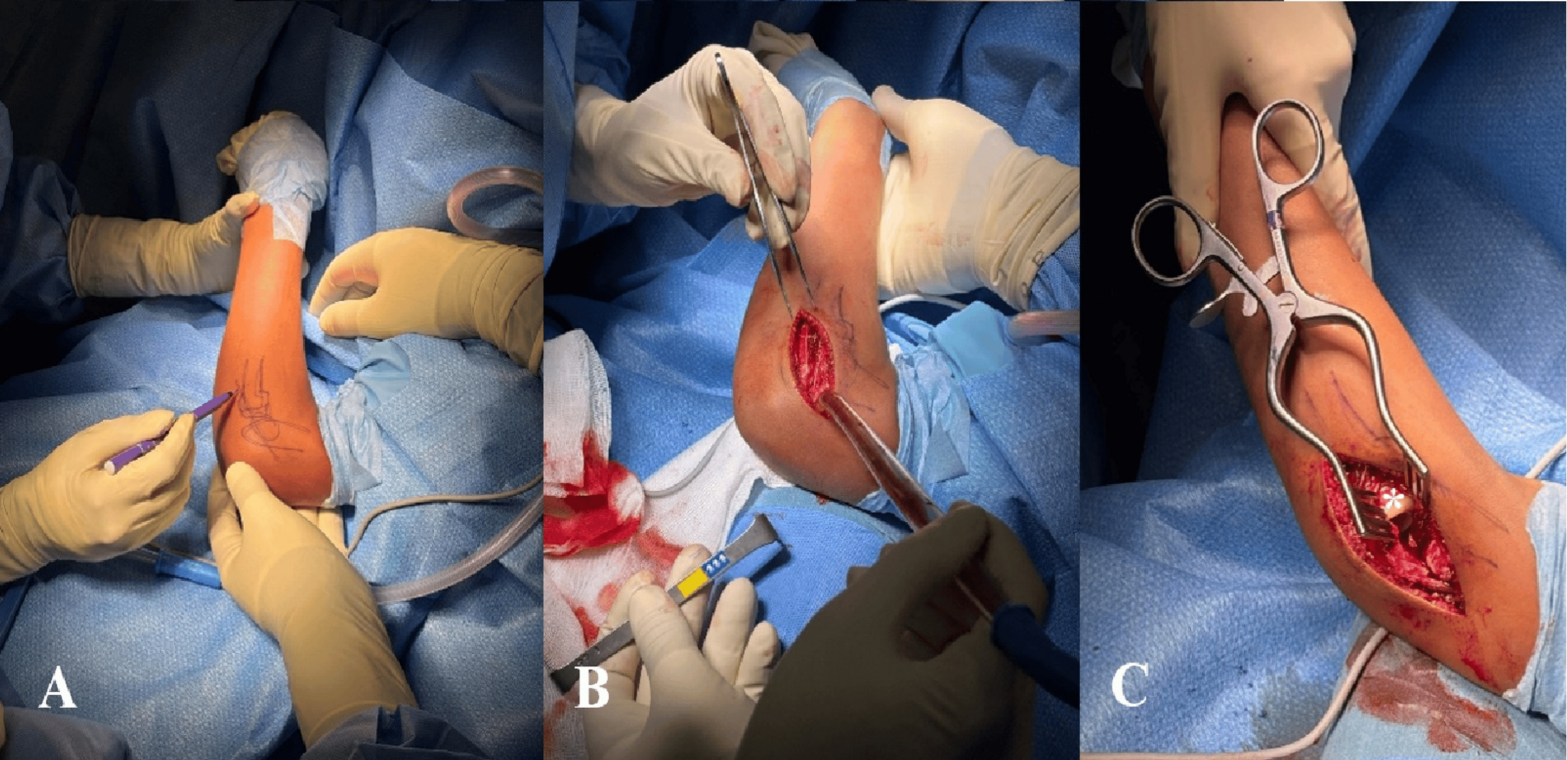
Lateral approach to the elbow through the Kocher interval A: Anatomical landmarks; B: Opening of the Kocher interval; C: Radiocapitellar joint exposed (radial head is marked with *).

**Figure 4:**
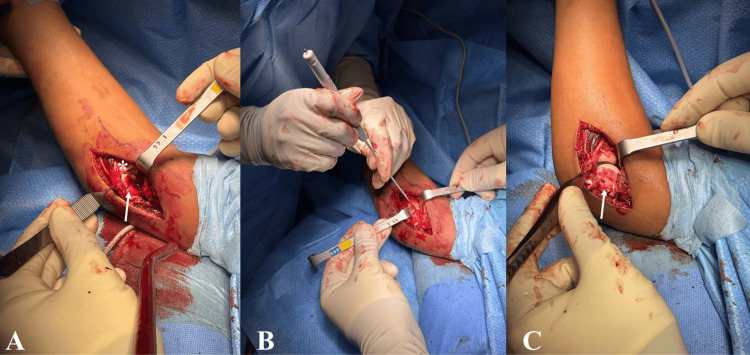
Open reduction and internal fixation A: Osteochondral fragment (white arrow, radial head is marked with *). B: Reduction and fixation with chondral darts (Arthrex®). C: Osteochondral fragment (white arrow).

The patient underwent surgical treatment under general anesthesia, in the supine position with the use of a tourniquet. Perioperative antibiotic prophylaxis with cefazolin for 24 hours was administered. The surgical approach chosen was a lateral elbow approach using the Kocher interval, with open reduction and internal fixation (ORIF) of the fragment using bioabsorbable darts from Arthrex® (Arthrex, Inc., Naples, Florida, United States). The patient was discharged the day after surgery. Postoperatively, the patient was immobilized in an above-elbow cast for three weeks. After this period, rehabilitation was initiated with good clinical progression, and the patient returned to sports activities three months after surgery (Figures 3-4).

**Figure 5:**
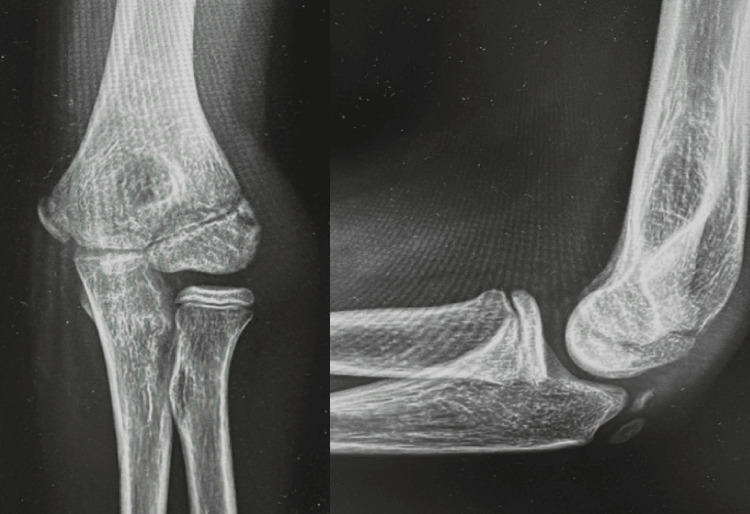
Left elbow radiographs: one-year postoperative follow-up Anteroposterior and lateral views of the elbow.

At one-year post-operative follow-up, the patient remains asymptomatic, with a full range of motion and radiographic evidence of fracture consolidation. No complications have been reported (Figure 5).

## Discussion

Capitellum fractures in the pediatric population represent a rare and frequently underdiagnosed entity, particularly in younger children, in whom a large portion of the distal humeral epiphysis remains cartilaginous, thereby limiting the diagnostic value of initial radiographic evaluation. As illustrated in our case, plain radiographs often fail to clearly demonstrate the fracture line, revealing only indirect signs such as a positive fat pad sign or small intra-articular fragments; this reinforces the need for a high index of clinical suspicion. This fracture pattern has been widely recognized in the literature and is included among TRASH lesions, as described by Waters et al. [1,9].

In recent years, multiple case reports and case series have emphasized CT scan and magnetic resonance imaging (MRI) as the most useful adjunctive imaging modalities to confirm the diagnosis and accurately characterize the fracture pattern in cases with a high index of suspicion [1,2,7,8]. In our hospital setting, a CT scan is particularly invaluable since it is more easily and readily available in the emergency department setting than an MRI. CT scans play an important role in fractures where the fragment is predominantly cartilaginous with a variable amount of subchondral bone, such as Kocher-Lorenz fractures [2,7,8].

In our patient, a Kocher-Lorenz fracture was identified, characterized by an osteochondral fragment with a thin layer of subchondral bone. This fracture pattern represents one of the most challenging lesions to detect on initial radiographic evaluation and to stabilize due to the limited amount of subchondral bone available for fixation. Regarding surgical technique, as reported in other recent studies, the preferred method is ORIF through a lateral approach using the Kocher interval, which allows adequate exposure of the capitellum while minimizing the risk of iatrogenic injury to neurovascular structures [1,8,10,12].

Regarding fixation methods, several options are available, including headless compression screws, absorbable Kirschner wires, and chondral darts [4,8,12]. Kang et al. retrospectively analyzed a cohort of 26 pediatric patients with Dubberley type IIA fractures and demonstrated that achieving stable fixation, whether using headless screws, absorbable wires, or compression darts, results in excellent functional outcomes with low complication rates and a rapid return to daily activities [13]. In our case, the use of bioabsorbable darts (Arthrex®) allowed for anatomical reduction and adequate stabilization of the fragment while avoiding the need for an additional surgical procedure for implant removal.

The postoperative evolution of our patient, characterized by complete fracture consolidation, full recovery of range of motion, and return to sports activities by the third postoperative month, is consistent with the outcomes reported in other studies. Late complications, such as stiffness or growth plate disturbances, are rare; even in Kocher-Lorenz fractures, traditionally associated with a higher risk of nonunion or fragment fragmentation, current literature supports that the prognosis is generally excellent when the diagnosis is made early and appropriate treatment with anatomical reduction is performed [4,8,10,11,14].

## Conclusions

Capitellum fractures in the pediatric population are rare injuries and may be easily underdiagnosed. Our case report highlights the importance of maintaining a high index of clinical suspicion in the emergency department setting, particularly when initial radiographs are inconclusive. In such cases, additional imaging modalities, including CT scan and MRI, may be helpful in establishing the diagnosis.

In our experience, early surgical management with anatomical reduction and stable fixation was associated with excellent clinical and functional outcomes, even in a challenging fracture pattern such as a Kocher-Lorenz fracture. While larger studies are required to define standardized treatment guidelines, timely diagnosis and careful surgical technique remain important considerations in the management of pediatric capitellum fractures.
